# Imbalance between HAT and HDAC Activities in the PBMCs of Patients with Ankylosing Spondylitis or Rheumatoid Arthritis and Influence of HDAC Inhibitors on TNF Alpha Production

**DOI:** 10.1371/journal.pone.0070939

**Published:** 2013-08-15

**Authors:** Eric Toussirot, Wasim Abbas, Kashif Aziz Khan, Marion Tissot, Alicia Jeudy, Lucile Baud, Ewa Bertolini, Daniel Wendling, Georges Herbein

**Affiliations:** 1 Clinical Investigation Center Biotherapy CBT-506, University Hospital of Besançon, Besançon, France; 2 Department of Rheumatology, University Hospital of Besançon, Besançon, France; 3 Department of Therapeutics, University of Franche Comté, Besançon, France; 4 UPRES EA 4266, Pathogens and Inflammation Laboratory, SFR FED 4234, University of Franche Comté, Besançon, France; 5 Department of Virology, University Hospital of Besançon, Besançon, France; INSERM-Université Paris-Sud, France

## Abstract

**Objective:**

Acetylation or deacetylation of histone proteins may modulate cytokine gene transcription such as TNF alpha (TNF). We evaluated the balance between histone deacetytlase (HDAC) and histone acetyltransferase (HAT) in patients with rheumatoid arthritis (RA) or ankylosing spondylitis (AS) compared to healthy controls (HC) and determined the influence of HDAC inhibitors (trichostatin A -TSA- or Sirtinol -Sirt-) on these enzymatic activities and on the PBMC production of TNF.

**Methods:**

52 patients with RA, 21 with AS and 38 HC were evaluated. HAT and HDAC activities were measured on nuclear extracts from PBMC using colorimetric assays. Enzymatic activities were determined prior to and after *ex vivo* treatment of PBMC by TSA or Sirt. TNF levels were evaluated in PBMC culture supernatants in the absence or presence of TSA or Sirt.

**Results:**

HAT and HDAC activities were significantly reduced in AS, while these activities reached similar levels in RA and HC. *Ex vivo* treatment of PBMC by HDACi tended to decrease HDAC expression in HC, but Sirt significantly reduced HAT in RA. TNF production by PBMC was significantly down-regulated by Sirt in HC and AS patients.

**Conclusion:**

HAT and HDAC were disturbed in AS while no major changes were found in RA. HDACi may modulate HDAC and HAT PBMC expression, especially Sirt in RA. Sirtinol was able to down regulate TNF production by PBMC in HC and AS. An imbalance between HAT and HDAC activities might provide the rationale for the development of HDACi in the therapeutic approach to inflammatory rheumatic diseases.

## Introduction

Rheumatoid arthritis (RA) and ankylosing spondylitis (AS) are inflammatory rheumatic diseases characterized by chronic inflammation located respectively on the peripheral joints and the axial skeleton. Active pro-inflammatory cytokines such as TNF alpha (TNF), IL-1beta or IL-6 are key players in the synovial inflammation of RA, and TNF is also considered to be a major cytokine driving inflammation in AS. Indeed, all these pro-inflammatory cytokines participate in a cascade of events, leading to synovial/sacroiliac cellular infiltration that causes pain, joint damage and progressive disability. Various factors may regulate cytokine production such as polymorphisms in the promoter region of cytokine gene, cytokine-cell interactions and therapeutic interventions. Accumulating evidence suggests that epigenetic modifications can modulate gene transcription and thus could regulate the production of pro-inflammatory cytokines [1]. The epigenetic phenomenon refers to different biochemical reactions on the DNA without changing its sequence, including histone modifications [2,3]. These changes in histone proteins involve various reactions such as methylation, ubiquitination, phosphorylation, sumoylation and acetylation. Acetylation is certainly the best characterized post-translational modification of core histones. Histone acetylation is catalyzed by histone acetyltransferase (HAT) while deacetylation is influenced by histone deacetylase (HDAC). It is accepted that acetylation of histones is associated with enhanced rates of gene transcription, while deacetylation represses gene expression by condensing the chromatin [3,4]. The ratio of HDAC on HAT may thus give insights into the rate of gene transcription. Four classes of HDAC have been identified: Class I HDACs (HDAC 1, 2, 3 and 8) are primarily located in the nucleus while Class II HDACs (HDAC 4,5,6,7,9) translocate from the cytoplasm to the nucleus. HDAC 11 is the only member of HDAC Class IV, and HDAC Class III, or sirtuins, is a distinct class of enzymatic activity dependent on NAD^+^ [5].

Histone deacetylase enzymes target histone proteins but also non-histone proteins. Indeed, HDAC activities can interfere with inflammatory signalling pathways, transcription factors and tumor suppressor genes, regulating cellular activation, proliferation and differentiation. Thus, HDAC inhibitors (HDACi) have been developed in cancer therapy in an attempt to inhibit cell proliferation and to induce cell cycle arrest or apoptosis [6]. However, besides these antineoplastic properties, HDACi have also been shown to display anti-inflammatory effects, ameliorating joint inflammation and regulating the production of a range of cytokines in animal models of RA [4,7].

The equilibrium between HDAC and HAT activities could thus be considered as a relevant marker for gene regulation, including those coding for pro-inflammatory cytokines such as TNF. There are limited results on the ratio of HDAC/HAT activity in patients with RA and other forms of arthritis including AS. In this study therefore, we aimed to evaluate the HDAC / HAT ratio in the peripheral blood of patients with RA or AS and to analyze the influence of HDACi on the production of TNF by peripheral blood mononuclear cells (PBMCs).

## Materials and Methods

### Patients

We enrolled 52 patients with RA meeting the 1987 American College of Rheumatology criteria and 21 patients meeting the modified New York criteria for AS. They were all receiving follow-up at the department of Rheumatology in the University Hospital of Besançon, France. In RA, disease activity and functional impairment were evaluated using the DAS28 score and Health Assessment Questionnaire (HAQ), respectively. Low disease activity was defined by a DAS28 < 3.2, moderate disease activity by a DAS28 ≥ 3.2 and ≤ 5.1 and high disease activity by a DAS28 > 5.1. For AS, clinical activity was evaluated using the Bath Ankylosing Spondylitis Disease Activity Index (BASDAI), the Ankylosing Spondylitis Disease Activity Score (ASDAS-CRP score) and the Bath Ankylosing Spondylitis Functional Index (BASFI). Erythrocyte sedimentation rate (ESR), C reactive protein (CRP) levels and circulating TNF were used as laboratory parameters to assess inflammation. Positivity for rheumatoid factors, anti-CPP antibodies and HLA-B27 status were also recorded. In the RA group, patients were receiving low dosage corticosteroids (< 10 mg prednisone daily) and/or traditional DMARDs (methotrexate, leflunomide, sulfasalazine or hydroxychloroquine). In the AS group, all patients received non-steroidal anti-inflammatory drugs (NSAIDs) and some of them had sulfasalazine or methotrexate. At the time of inclusion, no RA or AS patients were receiving TNF blocking agents or other biologics.

### Control subjects

The control group consisted of 38 healthy subjects without inflammatory conditions or treatment. All the patients and healthy controls (HC) gave their written informed consent to participate in the study according to the Helsinki declaration. The study protocol was approved by the local ethics committee (Comité de Protection des Personnes EST-2, study registered under the number 08/498).

### Peripheral blood mononuclear cell isolation and cell lysate preparation

PBMCs were prepared from the peripheral blood of patients and HC and were cultured in RPMI-1640 medium supplemented with 10% (v/v) pooled AB human serum (Sigma), as previously described [8]. Isolation of nuclear and cytoplasmic extracts was performed as previously described [9]. Cells were washed with wash buffer (10 mM HEPES, pH 7.6, 10 mM KCl, 2 mM MgCl_2_, 1 mM EDTA). Cell pellets were then incubated on ice with cytoplasmic isolation buffer (10 mM HEPES, pH 7.6, 10 mM KCl, 2 mM MgCl_2_, 1 mM EDTA, 0.02% NP-40). Cytoplasmic extracts were collected by centrifugation and the nuclear pellets were washed twice in wash buffer, spun, and incubated for 15 min on ice with nuclear isolation buffer (20 mM HEPES pH 7.6, 420 mM NaCl, 1.5 mM MgCl_2_, 0.2 mM EDTA, 25% glycerol). Supernatants containing nuclear extracts were collected by centrifugation and stored at -80° C. Protease inhibitors (1 mM DTT, 1 mM PMSF, 1 microg/ml aprotinin, 1 microg/ml leupeptin, 1 microg/ml pepstatin) were added to all solutions. Protein concentration in nuclear and cytoplasmic extracts was determined by the Bradford method using a BioPhotometer (Eppendorf, Hamburg, Germany).

### HAT and HDAC activities

The EpiQuik™ HDAC Activity Assay colorimetric kit (Epigentek, Farmingdale, NY) and the HAT activity assay kit (Enzo Life Sciences, Koropi, Greece) were used to evaluate HAT/HDAC activities in PBMC nuclear extracts. Global HDAC activity was evaluated. These activities were measured prior to and after *ex vivo* exposure of PBMCs to the following HDACi: trichostatin A (TSA), a Class I and Class II inhibitor (1 microM), and sirtinol (Sirt), a sirtuin inhibitor (10 microM). The use of concentrations of TSA up to 1 microM has been previously reported in studying the effect of HDACi on macrophages and monocytoid cells [10,11].

### Circulating TNF and production of TNF by PBMCs

Circulating TNF was evaluated in sera from patients and HC using an ELISA assay (Quantikine assay, R&D Systems, Minneapolis, MN). PBMCs from HC and from patients with RA or AS were treated or not with TSA (1 microM) or Sirt (10 microM), and TNF production in supernatants was measured by ELISA after one and three days of culture.

### Regulation of transcription from the human TNF (huTNF) promoter in the presence of HDACi

To assess the epigenetic regulation of the huTNF promoter, U937 cells (ATCC) were transiently transfected with 20 microg of a plasmid expressing the luciferase gene under the control of the huTNF promoter, pTNF-Luc (a gift from Dr D. Kwiatkowski, International Child Health Group, University Department of Pediatrics, John Radcliffe Hospital, Oxford, UK) [12] using electroporation system according to the manufacturer’s instructions (BioRad, Hercules, CA) [9]. Twenty-four hours later, the cells were treated with TSA (1 microM) or Sirt (10 microM). At 24 hrs post-treatment, luciferase activity was measured in cell lysates using a luminometer (TD-20/20; Promega, Madison, WI) as previously described [9]. Values normalized to protein concentrations were expressed in fold increase over unstimulated control values.

### Detection of apoptosis

PBMCs (2X10^6^/ml) were remained untreated or treated with TSA (1 microM) and Sirtinol (10 microM) for 24 or 72 h. The cells were washed in cold phosphate-buffered saline (1X PBS), supernatant was discarded and 10^6^ PBMCs were resuspended in 1 ml 1X annexin binding buffer. 5 microl of FITC labeled annexin V (BD Pharmingen™, San Diego, CA) was incubated in 100 microl of resuspended PBMCs. After 15 min incubation at room temperature in the dark, 400 microl of 1X annexin binding buffer was added and 10,000 cells were acquired by FACSCalibur with CELLQuest software (BD Biosciences, Mountain View, CA) [13].

### Cell viability assay

Cell viability was measured using the MTT assay kit (Cayman Chemical, AnnArbor, MI). PBMCs were seeded at a density of 0.1X10^6^/well in triplicates in 96-well plates in a final volume of 100 microl medium containing TSA (1 microM) or Sirtinol (10 microM). Cell culture medium without cells was used as a blank. After 24 hrs or 72 hrs, 10 microl MTT reagent was added to each well and the plates were incubated for another 4 h in the cell culture incubator at 37° C. The plate was centrifuged at 500 g for 10 min. The cell culture media was aspirated and 100 microl crystal dissolving solution was added to each well and the absorbance was measured at 570 nm using Multiskan Ex (Thermo Electron Corporation, France).

### Statistical analysis

Results were expressed as mean ± standard error of the mean (SEM). Non-parametric tests were used. Statistical analysis between the three groups (RA, AS and HC) involved non-parametric analysis of variance (ANOVA) using the Kruskal-Wallis test. This test was used to compare age, ESR, CRP and circulating TNF. The Mann Whitney test was used for comparing HAT activity (with and without HDACi), HDAC activity (with and without HDACi) and HDAC/HAT ratio between RA and HC and between AS and HC. The Wilcoxon test was used for the paired comparison of spontaneous HAT or HDAC activity and enzymatic activity under HDACi exposure, and for comparing TNF production in PBMC culture with and without HDACi. Qualitative data (gender) were analyzed using the Chi-squared test. A Spearman’s *r*-test was used to calculate correlations between HAT and HDAC activities and indices of AS or RA disease activity. Values of *p* less than 0.05 were considered significant.

## Results

### Clinical characteristics of the studied population

Patients with RA had mild disease activity (DAS28 > 3.2) while disease was more active in AS patients (BASDAI > 4 and ASDAS > 2.1). Most of the laboratory parameters of inflammation investigated (ESR, CRP and TNF) were elevated in RA and AS compared to HC (Table 1).

**Table 1:** Clinical and laboratory characteristics of the studied patients with rheumatoid arthritis, ankylosing spondylitis and healthy controls.

	**RA**	**AS**	**HC**	**P** (#)
N	52	21	38	
**Age** (years)				
(mean ± SEM)	56.9 ± 1.5	44.3 ± 3.2	34.6 ± 1.8	< 0.0001
**Disease duration** (years)				
(mean ± SEM)	11.4 ± 1.2	14.1 ± 2.2		
**Sex**				
M/F	16/36	18/3	12/26	< 0.0001
**DAS28**				
(mean ± SEM)	3.8 ± 0.2			
**HAQ**				
(mean ± SEM)	1.09 ± 0.1			
**BASDAI**				
(mean ± SEM)		4.5 ± 0.5		
**ASDAS-CRP**				
(mean ± SEM)		2.9 ± 0.3		
**BASFI**				
(mean ± SEM)		3.7 ± 0.6		
**ESR** (mm/h)				
(mean ± SEM)	23.8 ± 2.8	27.3 ± 6.5	7.8 ± 1.5	< 0.0001
**CRP** mg/L				
(mean ± SEM)	16.1 ± 5.8	20.4 ± 7.9	2.7 ± 0.6	< 0.0001
TNF pg/L				
(mean ± SEM)	7.9 ± 1.05	3.5 ± 0.8	4.9± 1.3	0.001
**Positive Rheumatoid factors**				
N and (%)	42 (81)			
**Positive anti CPP antibodies**				
N and (%)	34 (65.4)			
**HLA-B27**				
N and (%)		19 (90)		
**Treatments**				
**NSAIDs**				
N and (%)		17 (81)		
**Corticosteroids**				
N and (%)	35 (67)	1 (4.7)		
[mean dosage mg/day]				
	[6.4 ± 1.5]	[5]		
**MTX**				
N and (%)	28 (54)	1 (4.7)		
**DMARDs others**				
N and (%)	19 (36)	2 (9.5)		

RA: rheumatoid arthritis; AS: ankylosing spondylitis; HC: healthy controls; DAS28 : disease activity score 28 joints; HAQ: health assessment questionnaire; BASDAI: Bath ankylosing spondylitis disease activity index; ASDAS: ankylosing spondylitis disease activity score; BASFI: Bath ankylosing spondylitis functional index; ESR: erythrocyte sedimentation rate; MTX: methotrexate; #: Kruskall-Wallis test

### HAT and HDAC activities were reduced in patients with AS

**Figure 1:**
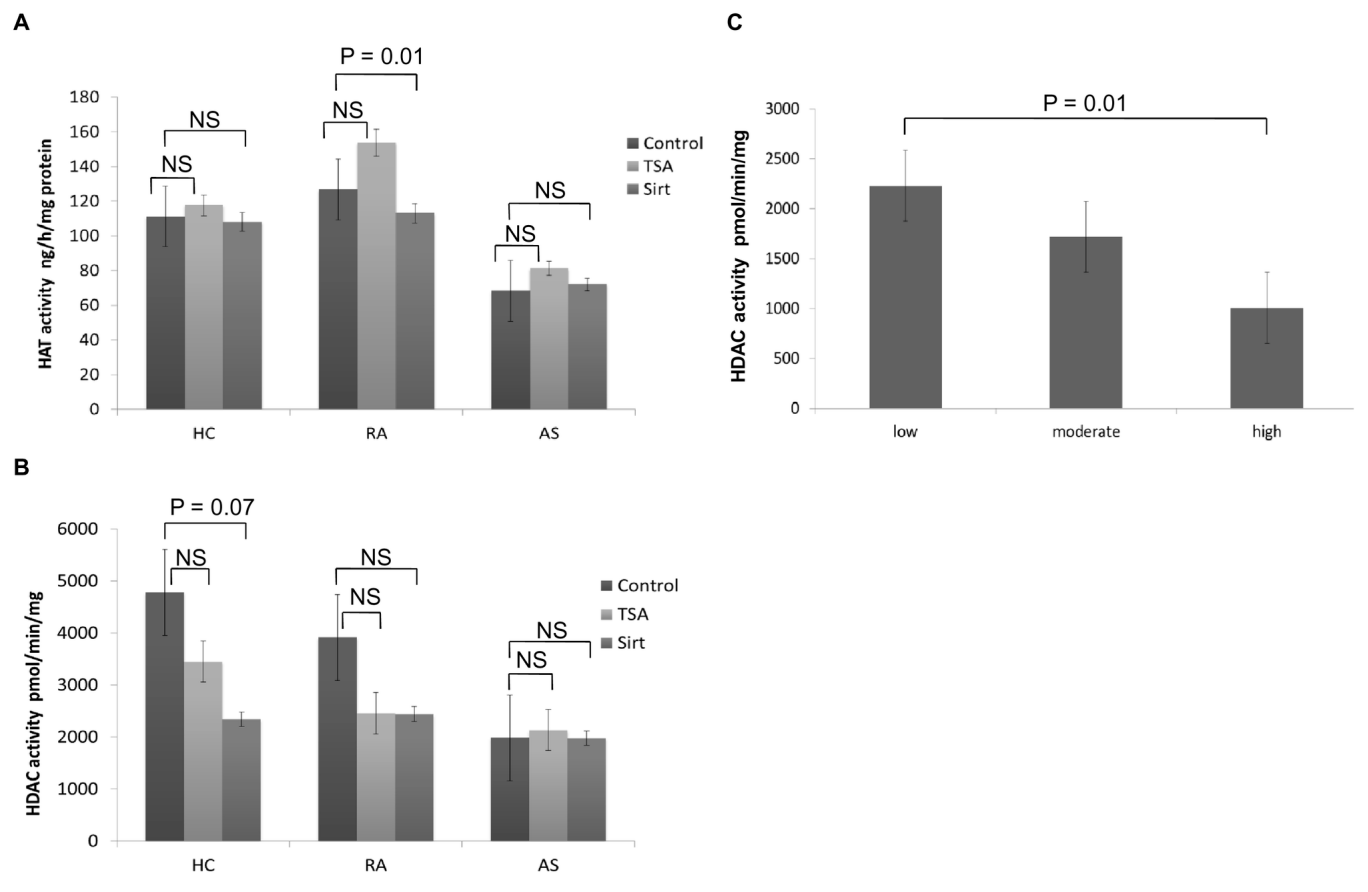
HAT and HDAC activity in HC, RA and AS subjects. Histone acetyltransferase activity (**A**) and histone deacetylase activity (**B**) in PBMC nuclear extracts from healthy controls (N = 38) and patients with rheumatoid arthritis (N = 52) or ankylosing spondylitis (n = 21). Enzymatic activity was measured without (control) and with histone deacetylase inhibitors, trichostatin A (TSA) or sirtinol (Sirt). (**C**) HDAC activity in PBMC nuclear extracts from subpopulations of RA patients based on DAS28 score (low: DAS28 < 3.2; moderate: DAS28 ≥ 3.2 and ≤ 5.1, high: DAS28 > 5.1). Results were expressed as mean ± SEM (HAT: histone acetyltransferase; HDAC: histone deacetylase; HC: healthy controls; RA: rheumatoid arthritis; AS: ankylosing spondylitis)..

HAT activity was lower in AS compared to HC (68.2 ± 8.1 *vs* 111.3 ± 15.5 ng/h/mg; p = 0.05) while it was higher in RA, but the difference was not significant (126.8 ± 16.4 *vs* 111.3 ± 15.5 ng/h/mg; p > 0.05) (Table 2, Figure 1A). For HDAC, both AS and RA had decreased levels of activity, and again the difference was only significant between AS and HC (AS *vs* RA *vs* HC: 1984.6 ± 249 *vs* 3915.9 ± 790.3 *vs* 4778.9 ± 752.3 pmol/min/mg; AS *vs* HC: p = 0.01; RA *vs* HC: p > 0.05) (Table 2, Figure 1B). When examining the balance between deacetylation and acetylation, the HDAC/HAT ratio decreased in both RA and AS compared to HC, but without significant differences (all p > 0.05). ESR and CRP did not correlate with HAT or HDAC levels. However, TNF weakly correlated with HDAC activity in AS patients (p = 0.02). Interestingly, in RA patients with high disease activity and compared to patients with low disease activity, we observed a decreased HDAC activity (1009.3 ± 113.1 vs 2230.7 ± 254.3 pmol/min/mg; p = 0.01) (Figure 1C), an enhanced HAT activity (215.2 ± 57.2 vs 115.6 ± 42.5 ng/h/mg; NS) and a decreased ratio of HDAC/HAT activity (13.1 ± 8.2 vs 23.7 ± 12.6; NS)**.**

**Table 2:** Histone acetyl transferase (HAT) and histone deacetylase (HDAC) activities in PBMC nuclear extracts from patients with rheumatoid arthritis, ankylosing spondylitis and healthy controls.

	**RA**	**AS**	**HC**	**P**
**HAT** (ng/h/mg)				
(mean ± SEM)	126.8 ± 16.4	68.2 ± 8.1	111.3 ± 15.5	RA *vs* HC:NS*
				AS *vs* HC: p=0.05*
**HDAC** (pmol/min/mg)				
(mean ± SEM)	3915.9 ±790.3	1984.6 ± 249.0	4778.9 ± 752.3	RA vs HC:NS*
				AS vs HC: p=0.01*
**Ratio HDAC/HAT**				
(mean ± SEM)	49.1 ± 16.7	35.6 ± 6.9	65.4 ± 16.7	NS*
**HAT + TSA** (ng/h/mg)				
(mean ± SEM)	153.2 ± 30.3	81.2 ± 9.9	112.6 ± 21	HAT *vs* HAT + TSA**:HC:NS
				RA: NS AS: NS
**HAT + Sirt** (ng/h/mg)				
(mean ± SEM)	113.1 ± 14.0	71.9 ± 6.2	107.9 ± 15.9	HAT *vs* HAT+ Sirt** HC:NS
				RA: p = 0.01
				AS: NS
**HDAC + TSA** (pmol/min/mg)				
(mean ± SEM)	2457.1± 316.4	2134.2 ± 426.3	3450.6 ± 709.1	HDAC *vs* HDAC + TSA** HC:NS
				RA: NS AS: NS
**HDAC + Sirt** (pmol/min/mg)				
(mean ± SEM)	2440.6 ± 446.4	1975.4 ± 313.2	2341.6 ± 242.7	HDAC *vs* HDAC + Sirt**
				HC: p = 0.07
				RA: NS AS: NS

Results were given as spontaneous activities and after *ex vivo* treatment of PBMCs by HDAC inhibitors (RA: rheumatoid arthritis; AS: ankylosing spondylitis; HC healthy controls; TSA: trichostatin A; Sirt: sirtinol) (* Mann- Whitney test; ** Wilcoxon test).

### HDACi tended to induce changes in HDAC levels in HC and RA but not in AS

In HC, the *ex vivo* addition of TSA or Sirt on PBMCs did not induce a change in HAT activity (all p > 0.05). Conversely, there was a reduction in HDAC levels that was more evident with Sirt (51.1% reduction), but the results did not reach significance (p = 0.07). In the RA group, TSA induced a non-significant increase in HAT, while there was a modest but significant decrease in this activity under Sirt (p = 0.01). In addition, a 37.7% reduction in HDAC was observed with TSA or Sirt without significance (all p > 0.05). In PBMCs from AS patients, neither HAT nor HDAC activities were influenced by HDACi exposure (all p > 0.05) (Figure 1A and 1B).

### Influence of HDACi on TNF production by PBMCs

**Figure 2:**
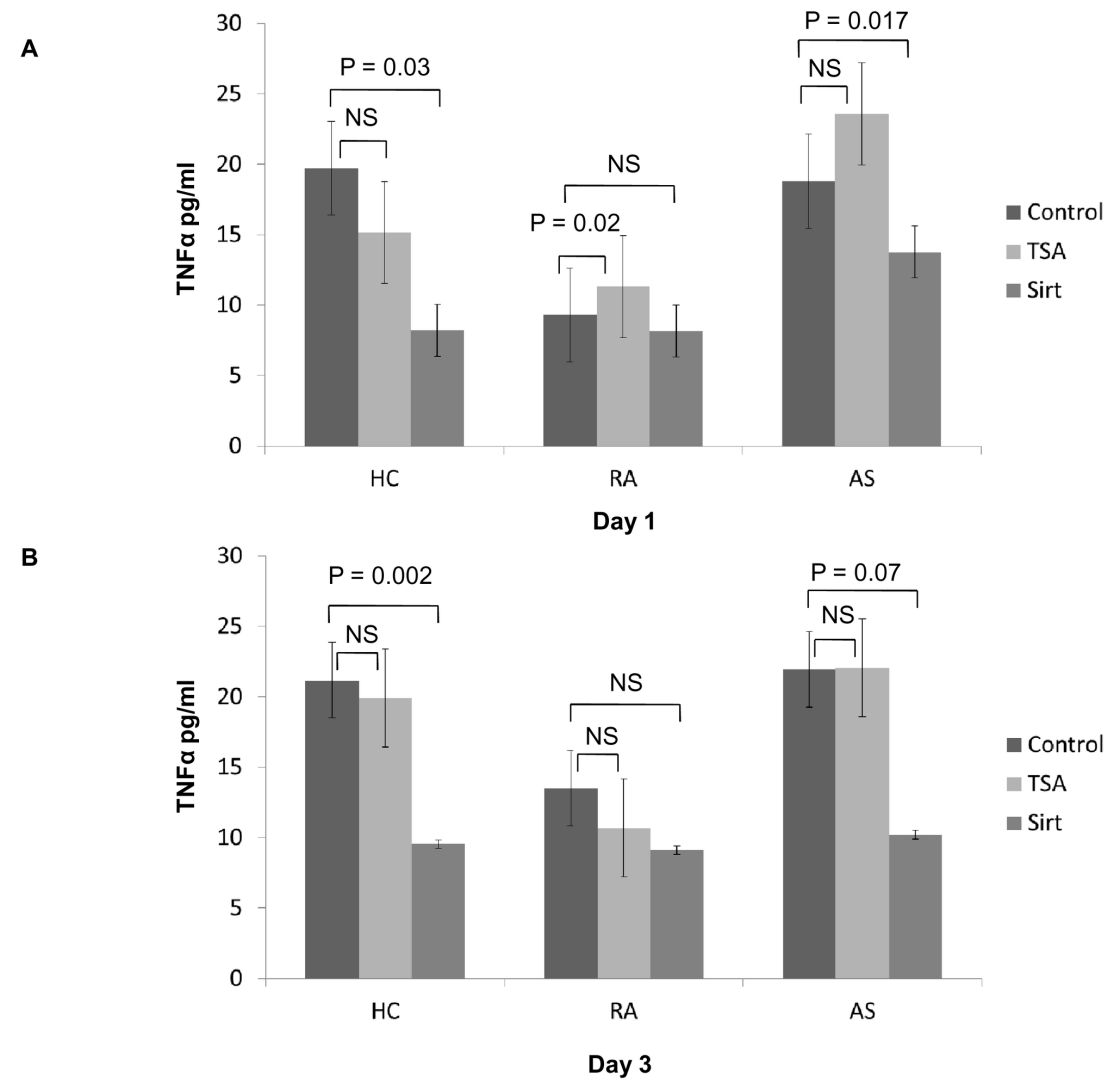
Quantification of TNF production in HC, RA and AS subjects. TNF production by PBMCs from healthy controls (N = 13) and patients with rheumatoid arthritis (N = 10) or ankylosing spondylitis (n = 21) after one (day one) (**A**) and three days (day three) (**B**) of cell culture. TNF was measured by ELISA in the culture supernatant without (control) and with histone deacetylase inhibitors: trichostatin A (TSA) or sirtinol (Sirt). Results were expressed as mean ± SEM (HC: healthy controls; RA: rheumatoid arthritis; AS: ankylosing spondylitis)..

**Figure 3:**
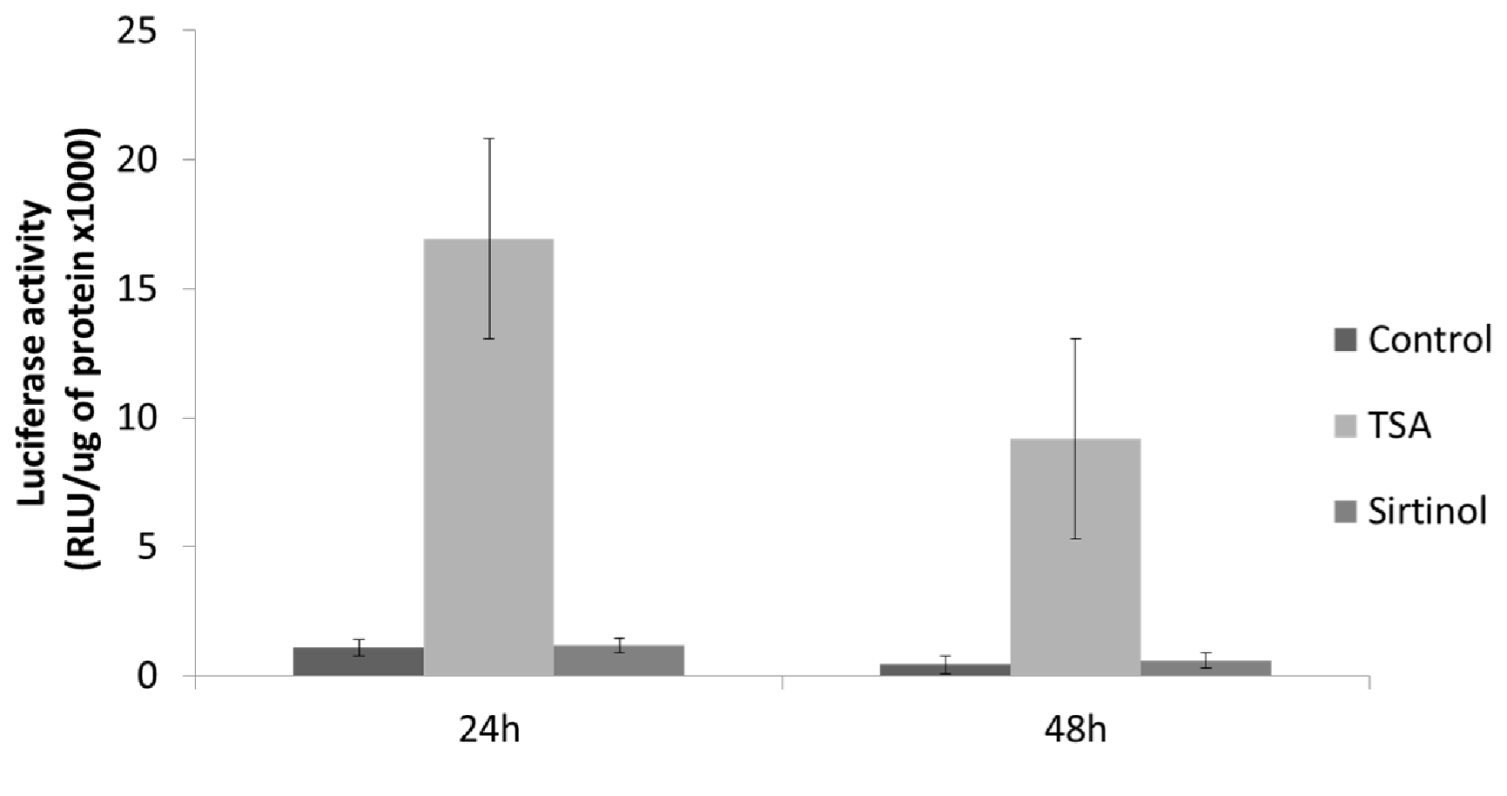
Activation of huTNF promoter by HDACi. TNF gene expression observed in the monocytoid U937 cells transiently transfected with a plasmid expressing the luciferase gene under the control of the huTNF promoter and treated without (control) or with histone deacetylase inhibitors: trichostatin A (TSA) or sirtinol (Sirt).

We then evaluated the influence of HDACi on TNF production by PBMC culture. In HC, TSA reduced TNF release but without significance at day one or three (all p > 0.05). Conversely, Sirt was more effective in reducing TNF production after one (p = 0.03) and three days (p = 0.002). In RA, these effects were less evident, with an increase in TNF at day one with TSA (p = 0.02) and a slight but non-significant decrease in TNF release at day three. Sirt had no effects on TNF production in RA. In AS, only Sirt induced a decline in TNF production at day one (p = 0.017) and three (p = 0.07) while TSA tended to induce TNF production at day one (Figure 2A and 2B). In parallel with this TNF production at day one post-treatment with TSA in RA and AS, there was higher TNF gene expression in the monocytoid U937 cells transiently transfected, with a plasmid expressing the luciferase gene under the control of the huTNF promoter and treated with TSA. Conversely, there was no variation in TNF gene expression in the same cells under Sirt treatment (Figure 3).

**Figure 4:**
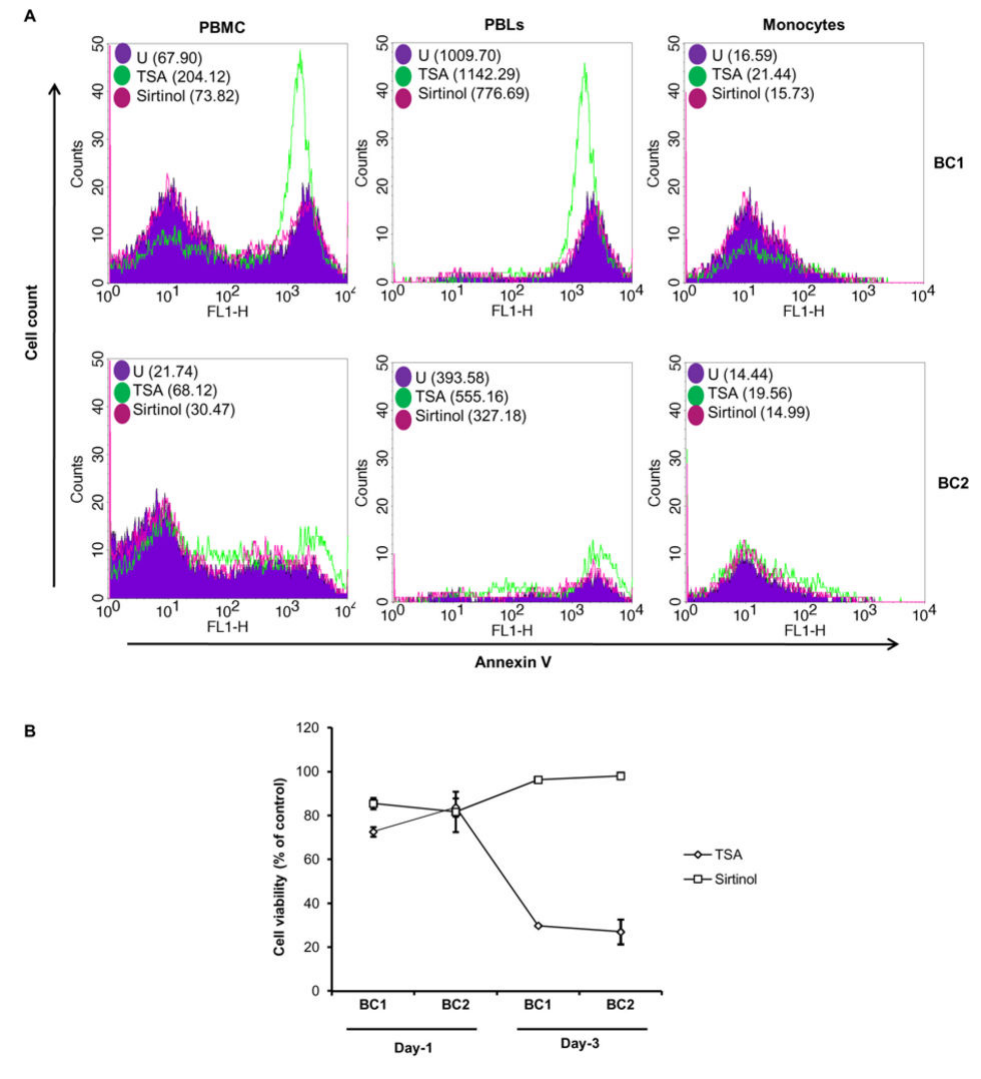
Effect of HDACi on cellular apoptosis and cell viability. **A**. Apoptosis of PBMCs, PBLs, and monocytes from healthy controls after treatment with TSA and Sirt. Apoptosis was measured by annexin-V assay as reported in the Materials and Methods chapter. Flow cytometric data at 72 h treatment are shown from two independent experiments. Mean fluorescence intensity (MFI) is indicated in brackets. BC, Buffy-coats of two healthy donors; PBLs: peripheral blood lymphocytes. **B**. Measurement of PBMC viability from healthy controls after one and three days of treatment with TSA and Sirt. Cell viability of PBMCs was determined by MTT assay after 24 h and 72 h treatment with TSA (1 microM) and sirtinol (10 microM). Data shown represent mean ± SD of triplicate wells.

***DetectionofapoptosisandcellviabilityinPBMCsfromhealthycontrols***: Apoptosis was then assessed in HC PBMCs treated with TSA or Sirt using annexin-V assay. TSA and to a much lesser extent Sirt triggered apoptosis in HC PBMCs, mostly in peripheral blood lymphocytes (PBLs), after 1 and 3 days of treatment (Figure 4A). In parallel, decreased viability of HC PBMCs using a MTT assay was observed, mostly with TSA treatment (Figure 4B).

## Discussion

Few studies have evaluated the equilibrium between HAT and HDAC activities in RA and there is no available data in AS [4,6,14]. This study was thus undertaken in order to clarify the balance between HDAC and HAT activities and to determine its influence on PBMC TNF production. Our results showed that HDAC/HAT activities were clearly disturbed in AS, with a significant reduction in both HAT and HDAC activities. In this patient group, the resulting balance in HDAC/HAT activities was also reduced compared to HC (but without significance) but this may be explained by the parallel decrease in both HAT and HDAC activities. By contrast, RA patients were characterized by a non-significant increase in HAT activity and a parallel decrease in HDAC, resulting in a reduced HDAC/HAT ratio. Interestingly, when we studied sub-populations of RA patients taken into account their DAS 28 score, we observed that RA patients with high disease activity displayed significant decreased HDAC activity, with a parallel but non significant enhanced HAT activity and decreased HDAC/HAT activity ratio. Limited data are available on the levels of HAT or HDAC activities in PBMC extracts from patients with RA. Gillespie et al. evaluated HAT and HDAC activities in PBMCs in a limited series of patients with RA (N = 8) and found increased HDAC activity but no changes in the levels of HAT compared to HC [15]. Besides these results, HAT or HDAC have been assessed in synovial tissue samples from RA patients. Conflicting results were reported, with decreased [16] or increased expression of HDAC [17,18] in synovial tissue from patients with RA compared to patients with osteoarthritis. These contradictory results may be explained in part by the disease duration of the patients evaluated and the treatment given, especially TNF blocking agents, and the biopsy samples evaluated (total synovium *versus* synovial fibroblasts). Low HDAC activity in RA synovial tissue was interpreted as a factor of hyperacetylation that can contribute to the activation of gene coding for pro-inflammatory cytokines and thus to the pathogenesis of RA [16]. Alternatively, high levels of HDAC (in PBMCs or synovial tissue) in RA may perpetuate inflammation by activating the RA synovial fibroblasts to produce mediators of joint damage [15,17,18]. In this sense, the levels of HDAC1 activity in synovial tissue correlated with the amount of cytoplasmic TNF [17]. It has also been reported that increased HDAC activity in synovial tissue may decrease expression of cell-cycle related molecules such as p16, p21 and p53 [18]. Presumably, the mechanisms linking HDAC activity to inflammatory mechanisms are mainly explained by non-histone modifications and by its influences on transcription factors [4,7,19]. For instance, it has been demonstrated that the subunits of the NF-kappaB signaling pathway, including the inhibitory transcription factor I-kappaB, are acetylated/deacetylated, and thus is influenced by the enzymatic couple HAT/HDAC [4,7]. High HDAC activity in this context may therefore maintain deacetylated such inhibitory factor allowing for NF-kappaB activation [19]. In AS, both HDAC and HAT were significantly reduced, with the resulting balance between HDAC and HAT also lower. This hyperacetylation status may also favor pro-inflammatory processes and ultimately contribute to AS pathogenesis.

In our study we also evaluated the *ex vivo* influence of HDACi on PBMC HAT/HDAC expression. Our results showed a decline, albeit not significant, in HDAC activity with TSA and Sirt in HC, while in RA, Sirt was able to reduce HAT. HDAC activity was also regulated in RA by TSA and Sirt, without reaching significance. By contrast, AS patients were not sensitive to TSA or Sirt. Trichostatin A is a pan-HDACi, inhibiting both Class I and Class II HDAC. We did not use specific HDACi in our study. HDAC1 and HDAC2 have been identified as the main HDAC Class I isoenzymes expressed within the synovium of patients with RA [17,18]. The NF-kappaB heterodimer composed of RelA/p65 and p50 proteins interacts with HDAC1 and HDAC2 [19]. Additionally, the localization of both RelA/p65 and sirtuin 1 proteins on the gene promoter suggests that sirtuin 1 may actively repress the gene expression by deacetylating RelA/p65 directly on chromatin [20], making the regulation of NF-kappaB-dependent genes even more complex. Finally, an imbalance of inflammatory and antiinflammatory macrophages has been reported in RA synovium [21]. The reestablishment of macrophage equilibrium by eliminating the proinflammatory macrophages could be an effective therapeutic approach for RA. Preclinical studies have shown the therapeutic efficacy of an anti-human death receptor 5 (DR5) antibody called TRA-8 in eliminating a subpopulation of inflammatory macrophages which produce high levels of cytokines, including TNF [21]. Thus, further data are needed to better understand HDACi influence on the regulation of HAT/HDAC in RA. Also, it would be more effective to target these specific HDAC activities by selective inhibitors in specific cell types, such as inflammatory macrophages.

It has previously been shown that HDACi may regulate cytokine production [4,7,22–24]. When evaluating the influence of HDACi on the production of TNF by PBMCs in our patients, we observed that Sirt was more effective compared to TSA for reducing TNF in HC. Since HDACi have been reported to trigger apoptosis [25–28] and thereby could influence TNF production in cell culture, we assessed apoptosis in HC PBMCs treated with TSA or Sirt using annexin-V assay. We observed that TSA and to a much lesser extent Sirt trigger apoptosis in HC PBMCs after 1 and 3 days of treatment. In agreement with these data, we also observed decreased viability of HC PBMCs using a MTT assay, mostly with TSA treatment. Although we cannot exclude that the observed decreased TNF production in our cultures is due to enhanced apoptosis triggered by HDACi, we believe that it cannot be explained primarily by cell apoptosis. First, the most important TNF decrease in our cultures is observed with Sirt treatment, although Sirt triggers only limited PBMC apoptosis and cell viability is quite high under Sirt treatment. Second, TNF is produced mostly by monocytes/macrophages rather than by PBLs, and we observed a preferential apoptosis of PBLs under HDACi treatment, as reported previously by others [25,26]. The reduction of TNF production was not significant in the RA group, while in AS, Sirt significantly reduced TNF production. The limited reduction of TNF production in our cultures could be explained by the fact that HDACi have only limited effects on immune cells not exposed to activating stimuli (for instance LPS) and their anti-inflammatory effects become apparent only after stimulation [26,29]. In agreement with this hypothesis, HDACi including TSA inhibit cytokine release (TNF, IL-6 and IL-12) by bone marrow derived macrophages exposed to microbial products and bacteria such as LPS and heat-killed *E. Coli* and *S. Aureus* [30,31]. Inhibition of TNF production has also been reported in PBMCs stimulated with LPS (10 ng/ml) one hour after preincubation with the HDACi SAHA [32]. Therefore, the *in vitro* activation of immune cells might have given a more pronounced effect of HDACi on TNF production.

In contrast to our data, Sirt has been reported to increase TNF release in both Kupffer cells and macrophages [33]. In our experiments we assessed the effect of Sirt on TNF production in human PBMCs and not in immortalized rat Kupffer cell line 1 (RKC1) or murine RAW264.7 macrophages. We tested human primary PBMCs that include monocytes and peripheral blood lymphocytes, and not purified rat or murine macrophage cell lines. Shen et al. assessed the production of TNF in cells treated with LPS, acetaldehyde and acetate while in our experiments PBMCs were not stimulated prior to the measurement of TNF production.

We observed in both AS and RA that TSA, but not Sirt, tended to induce TNF production at day one. These results are consistent with a preferential activation of the human TNF promoter by TSA in transfected U937 cells, indicating that TNF gene expression is repressed primarily by Class I and/or Class II HDACs. In fact, it has been reported that TNF gene expression is controlled by HDAC3, a Class I HDAC [34]. Again, we did not evaluate the change in TNF production with selective Class I or Class II HDACi. As stated above, targeting HDAC1 and/or HDAC2 isoenzyme activities is presumably more effective for modulating TNF in RA. A similar evaluation of cytokine regulation by HDACi has been performed by Gillepsie et al. who tested the influence of TSA and of a novel HDAC3 selective inhibitor (MI192) on cytokine production [15]. The PBMC production of interferon-gamma, IL-6 and TNF was regulated by these two agents, with a more pronounced response in HC compared to patients with RA. In our study, Sirt greatly suppressed TNF production in the AS group, and this sirtuin inhibitor thus seems an attractive agent for targeting this cytokine. Givinostat (originally called ITF2357) is a hydroxamic acid which inhibits class I and class II HDACs and reduces the production and release of proinflammatory cytokines including TNF, IL-1beta, IL-6, and interferon-gamma from human blood monocytes [27]. Givinostat exhibits significant therapeutic benefit in patients with systemic-onset juvenile idiopathic arthritis, particularly with regard to the arthritic component of the disease [24,35], and provide a clear rationale for further investigation in humans with chronic inflammatory conditions, such as RA and potentially AS.

In conclusion, we observed a disturbed balance of HDAC and HAT activity in our patients with AS and, to a lesser degree, in RA. *Ex vivo* exposure of PBMCs to HDACi could modulate these activities, mainly in HC, but also in RA. TNF production could be regulated by HDACi treatment, especially with Sirt in the AS group. The mechanisms explaining the anti-inflammatory effects of these HDACi are complex and certainly not limited to histone modifications, and a better understanding of the regulation of HDAC and HAT activities in AS and RA could lead to new therapeutic approaches in the future.
